# Lens density measurements by two independent psychophysical techniques

**DOI:** 10.1186/s40662-016-0054-6

**Published:** 2016-09-12

**Authors:** Anirbaan Mukherjee, Richard A. Bone

**Affiliations:** Department of Physics, Florida International University, 11200 SW 8th Street, Miami, 33199 FL USA

**Keywords:** Cataract, Heterochromatic flicker photometry, Lens optical density, Absolute threshold, Photopic, Scotopic

## Abstract

**Background:**

Cataract, a leading cause of vision impairment, is due to the lens becoming excessively optically dense. Change in the lens optical density (LOD) could be a useful indicator of incipient nuclear cataract and would necessitate the development of accurate measurement techniques.

Mapcat sf™ is a heterochromatic flicker photometer for measuring macular pigment optical density (MPOD) under photopic conditions. In the process, it also measures LOD that is needed in the calculation of MPOD. LOD is then converted by the instrument to “lens equivalent age” (LEA). However, varying cone photoreceptor ratios among individuals could affect the LEA measurement. Scotopic vision is mediated by rod photoreceptors; therefore, LEA measurement under scotopic conditions potentially provides a reliable standard for assessing other methods. The study was conducted to test the level of agreement between the LEA data obtained under photopic and scotopic conditions for a sample population. We also comment on factors that might contribute to any disagreement.

**Methods:**

LEAs were obtained by Mapcat sf for 25 subjects and compared with those obtained under absolute scotopic threshold conditions.

**Results:**

The mean scotopic LEA for the subjects was 2.7 years higher than the mean photopic LEA, but this difference was not statistically significant. Measurements by the two methods were reasonably correlated (r^2^ = 0.59, *p* < 0.0001). Significant individual differences in LEA by the two methods were found for six of the 25 subjects. Although our calculations included a standard long- to medium-wavelength-sensitive cone ratio, we found that different ratios could be found that rendered the differences in LEA insignificant for two of these six subjects. Variability in pupil diameter during scotopic measurements was considered another potential source of discrepancy between LEAs by the two methods.

**Conclusion:**

The absolute threshold technique, with long adaptation times, is probably impractical for routine lens density measurement, whereas Mapcat sf provided a rapid, straightforward test that may find its application in optometric/ophthalmic practice.

## Background

Mapcat sf™ is an optical instrument designed by one of the authors [1]. Its primary function is to measure macular pigment optical density (MPOD) in the human retina. In the process, it also measures the lens optical density (LOD) and uses this information to provide a necessary correction in the calculation of MPOD. In testing the instrument, we have come to the realization that it could be a useful adjunct in monitoring LOD, particularly in patients with incipient nuclear cataract. The present study was an attempt to validate the instrument’s capability of measuring LOD by comparing the results with those obtained using an absolute threshold technique.

The nucleus, cortex and posterior sub-capsule (PSC) are the structures of the lens that undergo degradative changes and, potentially, exhibit cataract [2, 3] However, the reasons for the changes are different for these three structures [4]. In the nucleus, conformational changes to the component protein molecules render them susceptible to cross-linking and aggregation to result in increased light scatter and reduced transparency [5]. This process is accompanied by the accumulation of fluorescent chromophores, rendering the nucleus yellow, brown or even blackish-brown. This is the most common type of cataract and is age-related. In the cortex, opacity is due to stress in the lens fibers [4] while in the PSC region it results from defective fiber production by the epithelium. PSC cataract has been attributed to diabetes, myopia, exposure to ionizing radiation and steroid intake [3]. Neither cortical nor PSC cataract is accompanied by optical density changes, rather by increased opacity (light scatter). Cataract, generally, is one of the leading causes of age-related vision impairment in the world [6]. Cataract can also develop in younger individuals due to genetic factors [7] and other factors from which one can safeguard, such as UV exposure [8], smoking [9] and obesity [10].

An accelerated increase in the optical density of the lens (lens yellowing) is an indicator of progression towards nuclear cataract. Ophthalmologists and optometrists typically grade yellowing and sclerosis on a 1 to 4 scale [11]. Alternatively, or in addition, a quantitative measurement of an individual’s LOD by a rapid, non-invasive test would be valuable [12, 13]. Examples of non-invasive, direct measurement techniques for measuring LOD are the signal-based autofluorescence [14] and the image-based reflectance [15] techniques. The technique of autofluorescence, aside from being expensive, is hampered by the scattering of light by the aging lens [16]. Reflectance techniques could potentially be compromised by the presence of drusen in the retina, particularly in older individuals [16]. On the other hand, psychophysical techniques involving either photopic or scotopic vision pose minimal risk and suffer no detrimental loss of signal due to scattering from the aging lens.

Techniques employing photopic vision depend on the responses of the cone cells, and are fast and easy. Such techniques, whether they are heterochromatic flicker photometry (HFP) [17] or color-matching, employ the photopic luminous efficiency function, which is dependent on the relative weighting of long (L)- and medium (M)-wavelength-sensitive cones [18]. Since the weights of the L and M cones are known to vary over a wide range among individuals, the reliability of data obtained from photopic methods is questionable. This point was addressed by Wooten et al. [12] who, in addition to measuring LOD under scotopic conditions (see below), used HFP to measure LOD under photopic conditions. Mapcat sf uses photopic viewing conditions to obtain MPOD and LOD [1]. The LOD is interpreted through a model proposed by Sagawa and Takahashi, 2001 [19] as “lens equivalent age” (LEA). Sagawa and Takahashi claimed that the variation in the luminous efficiency with age, at least in the short wavelength region of the spectrum, is due to the progressive yellowing of the lens.

Psychophysical methods employing scotopic vision, which is mediated by the rod cells, have been used to measure LOD [20, 21]. The measurements were made by comparing the scotopic spectral sensitivity functions of phakic and aphakic individuals. Differences in the functions were attributed to the transmittance of the lens in phakic individuals. In addition, the scotopic spectral sensitivity for aphakic individuals bears a marked resemblance to the rhodopsin absorbance spectrum. Therefore, one can compute the LOD spectrum by comparing the rhodopsin absorbance spectrum with an individual’s scotopic spectral sensitivity function obtained at absolute threshold [22]. More recent studies employing scotopic vision to measure LOD have been reported by Wooten et al. [12], Teikari et al. [17], and Najjar et al. [23]. Unlike the photopic case, with the problem of variation in the L and M cone weighting, rod-mediated vision is dependent on one template, the absorbance spectrum of the rod photopigment, rhodopsin. Making measurements under absolute scotopic threshold conditions requires long durations of dark adaptation time (DA), however, such methods yield results that are in agreement with those obtained by *ex vivo* techniques [12]. Of note, Najjar et al. [23] have reported that LODs measured under reduced dark adaptation time were in good agreement with those measured under extended periods of DA. Nonetheless, in order to test the validity of LOD measurements made by the Mapcat sf, we decided to compare the results with those obtained under absolute scotopic threshold conditions in the same individuals.

## Theory

### Photopic measurements

Mapcat sf, a research-grade instrument employing HFP, whose primary purpose is to measure MPOD, was used to measure LOD. In HFP, lights of two different wavelengths are presented in anti-phase with each other, first in the fovea and then in the parafovea or perifovea, and the relative intensities are adjusted for a flicker null or minimum. The flicker null occurs when the luminances of the lights are equal [24]. The MPOD spectrum has peak absorbance at 460 nm, decreasing almost to zero absorbance at around 550 nm [25, 26]. Hence to measure MPOD using HFP, the two lights should have peak wavelengths close to these values. Mapcat sf employs a blue LED (peak wavelength 455 nm, 3 W power) and a green LED (peak wavelength 515 nm, 1 W power). The choice of 455 nm was dictated by the availability of LEDs in this region of the spectrum. The use of 515 nm, rather than a value closer to 550 nm, together with the wide bandwidths of both LEDs, meant that the LED spectra had to be included in the calculation of MPOD at 460 nm as well as LOD which the Mapcat sf reports at 420 nm (see equations 1–4 below). For the foveal and perifoveal measurements, centrally viewed 1.5 and 15° diameter circular stimuli, respectively, are used. For the perifoveal measurement, the subject seeks to minimize flicker around the periphery (7.5° eccentricity) where the effect of macular pigment is negligible. In both cases, the intensity of the blue LED is adjusted by the subject by altering the frequency of fixed amplitude, 10 μs wide pulses in the kilohertz range. The intensity of the green LED is held constant and provides a stimulus luminance of ~ 20 cd/m^2^ that is comfortable for the subject (not glaring) yet sufficient to eliminate rod participation. The lens absorbs strongly in the short wavelength region of the visible spectrum [27] decreasing to almost zero absorbance at about 550 nm [28]. Therefore, the luminances of the two lights will be differentially affected by the lens in the perifoveal measurement (and by the lens and macular pigment in the foveal measurement).

Mapcat sf is a microprocessor-controlled instrument, with the blue and green LED intensities detected by a photodiode. The outputs of the photodiode at the perifoveal flicker null are given by

1 $$ {\phi}_{BP}={k}_P\kern0.28em {\displaystyle \int {I}_B}\kern0.28em \left(\lambda \right)\kern0.28em S\kern0.28em \left(\lambda \right)\kern0.28em d\kern0.28em \lambda $$

2 $$ {\phi}_G={\displaystyle \int {I}_G}\kern0.28em \left(\lambda \right)\kern0.28em S\kern0.28em \left(\lambda \right)\kern0.28em d\kern0.28em \lambda $$

*I*_*B*_ and *I*_*G*_ are the intensity spectra of the blue and the green LEDs as measured by a spectrophotometer (Ocean Optics^®^). *S* (*λ*) is the spectral sensitivity of the photodiode. The multiplier *k*_*P*_ in Eq. (1) is the adjustment factor for the intensity of the blue LED needed by the subject to achieve the flicker null/minimum. Since luminances of the blue and green lights are equal at the flicker null,

3 $$ {k}_P=\frac{{\displaystyle \int {I}_B\kern0.28em \left(\lambda \right){V}_{10}\kern0.24em \left(\lambda, a\right)\kern0.28em d\kern0.28em \lambda }}{{\displaystyle \int {I}_G\kern0.28em \left(\lambda \right)\kern0.28em {V}_{10}\kern0.24em \left(\lambda, a\right)\kern0.28em d\kern0.28em \lambda }} $$

where *V*_10_ (*λ*, *a*) is the standard CIE 10° photopic luminosity function, *V*_10_ (*λ*), adjusted for age, *a*, according to the model of Sagawa and Takahashi [19]. Their model provides the average change in log luminous efficiency per year of age which, at the shorter wavelengths relevant for the Mapcat sf, they attributed to age-related changes in LOD. In choosing a 10° photopic luminosity function (the largest field size for which data are available), the effects on the function of macular pigment absorption are minimal, however, we had to assume that there would be negligible differences between *V*_10_ (*λ*) and a corresponding 15° luminosity function. From Eqs. 1, 2, and 3,

4 $$ \frac{\phi_{BP}}{\phi_G}=\frac{{\displaystyle \int {I}_G\kern0.28em }\left(\lambda \right)\kern0.28em {V}_{10}\kern0.28em \left(\lambda, a\right)\kern0.28em d\kern0.28em \lambda }{{\displaystyle \int {I}_B\kern0.28em \left(\lambda \right)\kern0.28em {V}_{10}\kern0.28em \left(\lambda, a\right)\kern0.28em d\kern0.28em \lambda }}\times \frac{{\displaystyle \int {I}_B\kern0.28em \left(\lambda \right)}\kern0.28em S\kern0.28em \left(\lambda \right)\kern0.28em d\kern0.28em \lambda }{{\displaystyle \int {I}_G\kern0.28em \left(\lambda \right)}\kern0.28em S\kern0.28em \left(\lambda \right)\kern0.28em d\kern0.28em \lambda } $$

**Fig. 1:**
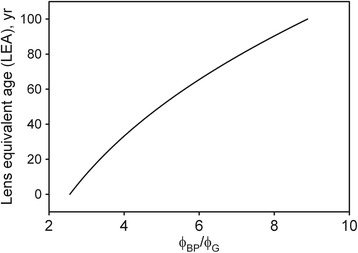
LEA as a function of the ratio of photodetector outputs for the perifoveal flicker null setting

Eq (4) was solved numerically by calculating $\frac{\phi_{BP}}{\phi_G}$ for a range of values of *a*. The result is the smooth curve shown in Fig. 1. The curve was programmed into the Mapcat sf microprocessor in order to compute a value of *a* from the subject’s perifoveal settings, *ϕ*_*BP*_ and *ϕ*_*G*_. We refer to *a*, which may well be different from the subject’s biological age, as the “lens equivalent age,” LEA. This is consistent with the well-known inter-individual variability in LOD at any given age [23]. We did find (see Results) that, on average, the LEA was close to the biological age, thereby giving us confidence in using the Sagawa and Takahashi [19] model.

### Scotopic measurements

Scotopic vision occurs under ambient light conditions below −2.5 log Trolands and is mediated purely by the rod cells. The spatial density of rods increases with eccentricity in the retina, going from zero in the foveal center to a peak of approximately 190,000 rods/mm^2^ at 20 to 30° from the fovea. Therefore, absolute threshold measurements require a parafoveal or perifoveal stimulus that is only affected by absorption in the lens and negligibly by the macular pigment [29]. Thus, the scotopic luminosity function at eccentricities above ~5° is affected primarily by the spectral transmittance, *T*_*L*_ (*λ*), of the lens and the rhodopsin absorbance spectrum, *R* (*λ*) [30].

5 $$ {V}_{scotopic}=R\;\left(\lambda \right)\;{T}_L\;\left(\lambda \right) $$

The lens transmittance was calculated from that of a 32-year-old, using a model proposed by van de Kraats and van Norren [31], and then modified for age using the template by Sagawa and Takahashi [19]. Absolute thresholds were obtained for each of the two stimuli located at the same retinal eccentricity used in the Mapcat sf perifoveal test and illuminated by LEDs of similar wavelengths. The luminances of the LEDs were varied using a neutral density wedge in order to achieve the absolute threshold condition. The corresponding luminances, *L*_*B*_ and *L*_*G*_ of the stimuli due to the light from the blue and green sources are

6 $$ {L}_B={\displaystyle \int B\kern0.28em \left(\lambda \right)\kern0.28em {T}_F\kern0.28em \left(\lambda \right)\kern0.28em {T}_w\kern0.28em \left({x}_b,\kern0.28em \lambda \right)\kern0.28em R\left(\lambda \right){T}_L\kern0.28em \left(\lambda, a\right)\kern0.28em d\lambda } $$

and

7 $$ {L}_G={\displaystyle \int G\kern0.28em \left(\lambda \right)\kern0.28em {T}_F\kern0.28em \left(\lambda \right)\kern0.28em {T}_w\kern0.28em \left({x}_g,\kern0.28em \lambda \right)\kern0.28em R\left(\lambda \right){T}_L\kern0.28em \left(\lambda, a\right)\kern0.28em d\lambda } $$

*B* (*λ*), *G* (*λ*) are the normalized intensity spectra of the blue and green LEDs obtained with an Ocean Optics^®^ spectrometer. *T*_*F*_(*λ*) is the transmittance of an auxiliary neutral density filter placed in the light path to achieve the necessary scotopic conditions. *T*_*w*_ (*x*_*b*_, *λ*) and *T*_*w*_ (*x*_*g*_, *λ*) are the transmittances of the variable density wedge at translational settings of *x*_*b*_ and *x*_*g*_, the positions at which the absolute threshold is detected for the blue and the green stimuli, respectively. At the absolute threshold, the luminance values for the test stimuli are equal, hence,

8 $$ \frac{1{0}^{{\textstyle \hbox{-} }1.2036{x}_g}}{1{0}^{{\textstyle \hbox{-} }1.1364{x}_b}}=0.59\kern0.28em \frac{{\displaystyle \int B\kern0.28em \left(\lambda \right)}\kern0.28em {T}_F\kern0.28em \left(\lambda \right)\kern0.28em {T}_w\kern0.28em \left({x}_b,\kern0.28em \lambda \right)\kern0.28em R\kern0.28em \left(\lambda \right)\kern0.28em {T}_L\kern0.28em \left(\lambda, a\right)\kern0.28em d\kern0.28em \lambda }{{\displaystyle \int G\kern0.28em \left(\lambda \right)}\kern0.28em {T}_F\kern0.28em \left(\lambda \right)\kern0.28em {T}_w\kern0.28em \left({x}_g,\kern0.28em \lambda \right)\kern0.28em R\kern0.28em \left(\lambda \right)\kern0.28em {T}_L\kern0.28em \left(\lambda, a\right)\kern0.28em d\kern0.28em \lambda } $$

**Fig. 2:**
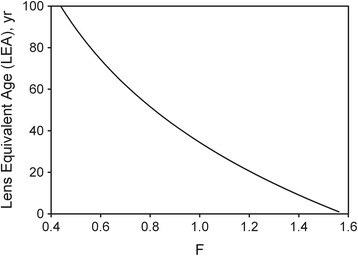
LEA as a function of F (Eq. 8). F itself is a function of the wedge settings required to achieve absolute threshold

In Eq. (8), the numerical factors 1.2036, 1.1364 and 0.59 are associated with the calibration of the neutral density wedge and the photodetector. All the wavelength-dependent functions are known; thus the right-hand side can be evaluated as a function of the LEA, *a*. Labeling that function as *F*, we calculated its value for various values of LEA, as shown in Fig. 2, and then modeled the relationship using a Sigmaplot^®^ curve-fit routine:

9 $$ LEA=\frac{a+bF}{1+cF+d{F}^2} $$

This rational four-parameter equation was found to give an excellent fit to the curve in Fig. 2 (R^2^ = 0.9998), thereby providing an accurate means of calculating the value of LEA from the value of F.

## Methods

### Instrument design

**Fig. 3:**
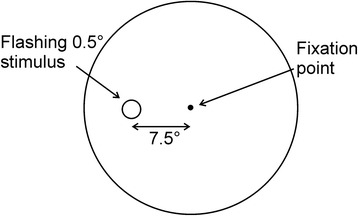
Visual field for absolute threshold measurements

The visual field produced by the instrument is shown in Fig. 3. Offset from the center at 7.5° eccentricity is a 0.5° stimulus illuminated by either a blue or green LED of peak wavelength 455 or 515 nm, respectively. The wavelengths and retinal location of these stimuli are very close to those used in the Mapcat sf perifoveal test. We reasoned that small amounts of macular pigment that might be present at 7.5° eccentricity would introduce a similar error in the measurement of LEA by each method. Additionally, rod density is relatively high at this eccentricity. In a similar study by Wooten et al. [12], wavelengths of 406 and 550 nm, derived from a monochromator, were selected to maximize the difference between the corresponding LODs. Correspondingly, the lens density template could be fit to the data with greater certainty. LEDs of wavelength 410 to 420 nm are certainly available, but their spectra extend down to about 370 nm where uncertainties in the rhodopsin absorbance and LOD are large. The intensity of the LEDs can be controlled, as in the Mapcat sf, by varying the frequency of fixed amplitude, 10 μs wide pulses in the kilohertz range. Such a control system, together with mounting the LEDs on substantial heat sinks and employing forced air cooling to minimize temperature changes, was found to prevent any measurable shift in the peak wavelength as the intensity was changed. The LEDs are cycled on (0.5 s) and off (1.0 s) resulting in the visual perception of short flashes that, when above threshold, were found easy to count. Similar flash durations have been used elsewhere [32]. At the center of the visual field is a small 0.25° fixation light illuminated by a low intensity red LED.

**Fig. 4:**
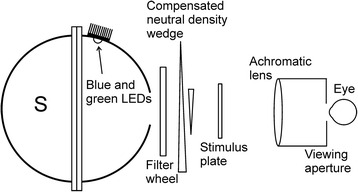
Optical system for measuring LEA under scotopic conditions

The optical layout of the instrument is shown in Fig. 4. The test LEDs are mounted in the upper interior part of an integrating sphere S. This arrangement results in spatially uniform light emerging from the front aperture of the sphere. In order to achieve scotopic conditions, a series of neutral density filters and a variable neutral density wedge filter are placed between the sphere S and the eye-piece. The wedge position is adjusted electromechanically via a stepper motor that is controlled by the operator. The pulse frequency for each LED, and therefore its intensity, is set at the lowest available level (~8 kHz), thereby reducing the likelihood of temperature-related wavelength shifts, and stimulus luminance is controlled entirely with the wedge. The housing for the eyepiece has an opaque screen at the left end in which precisely cut apertures define the size of the stimulus and fixation target. A dim red LED is mounted just behind the aperture for the latter. At the right end of the housing is an achromatic lens that can be moved back and forth to allow individual subjects to focus on the stimulus. A non-limiting viewing aperture is positioned so that the distance between the nodal point of the subject’s eye and the lens is equal to the focal length of the lens. It can be shown that such an arrangement maintains the same angular size and eccentricity of the stimulus for all subjects, regardless of adjustments to the lens position [1].

### Subject recruitment

Twenty-five subjects with no (self-reported) visual pathologies or color vision defects, and in the age range of 20 to 75 years (average age 39 ± 14 years), were recruited for the study from the University faculty, staff and students. Potential subjects were excluded from the study if they were unable to adjust the focusing optics to obtain sharply focused images of the stimuli on their retinas. Written informed consent was obtained from all subjects and the study was approved by the Florida International University’s Institutional Review Board.

### Photopic testing

Since there is generally a high level of agreement between the LOD in the left and right eyes of healthy subjects [12], only the right eye was tested. At the beginning of the test, the subject’s left eye was covered by an eye patch to minimize visual distractions. Since the optics of the instrument are enclosed in an instrument case, the room lighting was left on. The Mapcat sf was positioned on an adjustable table. The subject was seated in front of the viewing aperture and the operator adjusted the heights of the table and a chin rest to facilitate optimum comfort. The subject was then instructed to bring the 1.5° stimulus into sharp focus via a hand-held control. The flicker frequency was adjusted by the operator to 24 Hz, sufficiently high to ensure mediation of detection by the luminance channel. The subject was directed to fix his/her gaze at the center of the cross-hairs in the 1.5° stimulus and to adjust the intensity of the blue LED using the hand-held control until a flicker null/minimum was obtained. Once the subject reported a flicker null, the intensity value was recorded by the operator. A total of five settings were recorded with random offsets being introduced automatically by the instrument after each recording.

For the perifoveal phase of the test, the 1.5° stimulus was replaced by the 15° stimulus. The operator adjusted the flicker frequency to 31 Hz and instructed the subject to keep his/her gaze fixed at the center of the cross hairs and minimize flicker in the periphery of the stimulus by again adjusting the blue LED intensity. Five repeat measurements were again recorded. The built-in microprocessor then calculated the MPOD, the percentage of the blue light blocked by the macular pigment, the LOD and the LEA together with the associated standard errors.

### Scotopic testing

Scotopic testing of a subject was conducted within a few weeks at most after photopic testing. All subjects completed the tests over a 3-month period. Prior to dark adaptation, the subject adjusted the position of the eye-piece lens in order to bring the field of view into sharp focus. This was followed by a dark adaptation period of 30 min in complete darkness. As an additional precaution, the right test eye was covered with an eye patch. At the end of this period, the operator turned on the flashing blue stimulus and instructed the subject to fixate on the minimally lit red LED so that the stimulus was perceived peripherally. Initially, the stimulus intensity was set well above threshold so that the subject could see 14 flashes during a 20 s period. The intensity was then lowered incrementally by moving the neutral density wedge and, at each step, the subject reported the number of flashes that could be seen during the 20 s observation period. This is a modified version of the method of constant stimuli described in a similar study by Hammond et al. [33]. In the present study, when the number of flashes that could be perceived reached approximately 2 out of the 14 presented, the test was terminated. The operator then replaced the blue stimulus with the green one, and the test was repeated.

## Results

Photopic testing of a subject with the Mapcat sf required ~10 min. This included subject training using a PowerPoint presentation of the testing procedure, and the actual test. Scotopic testing on the other hand required almost an hour. This included subject training, 30 min for dark adaptation, and the test itself. The general feedback from the subjects was that they found the photopic testing easier than the scotopic testing. Within the photopic test, they reported that the perifoveal adjustments were easier than the foveal ones. This was evident from the standard deviations of the five flicker null settings. On average, the standard deviation for the perifoveal test was approximately half that for the foveal test.

The data obtained from scotopic measurements were used to generate scatter plots of the number of counts vs. wedge position, and a three-parameter sigmoidal function, shown in Eq. (10), was fit to the data.

10 $$ N=\frac{a}{1+{e}^{-\frac{\left(x\mathit{\hbox{-}}{x}_0\right)}{b}}} $$

**Fig. 5:**
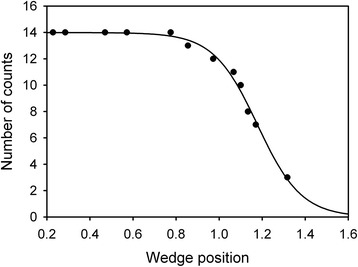
Representative scatter plot for the blue stimulus of number of flashes detected in a 20 s period as a function of wedge position. An inverse sigmoidal curve of the form in Eq. (10) was fit to the data to obtain the absolute threshold

*N* is the number of counts and *x* is the neutral density wedge position. A representative graph is shown in Fig. 5. From the fit curve, we used a 50 % visibility criterion to define the absolute threshold wedge setting, represented by the parameter *x*_*o*_ in Eq. (10). This is the wedge setting for which 50 % of the flashes were visible. By substituting threshold wedge settings for the blue and green stimuli into the left side of Eq. (8), we obtained the quantity F, and substituting this into Eq. (9) gave us the LEA. The uncertainty in LEA was calculated from the uncertainty in the appropriate parameter, *x*_*o*_, in the sigmoidal function.

**Fig. 6:**
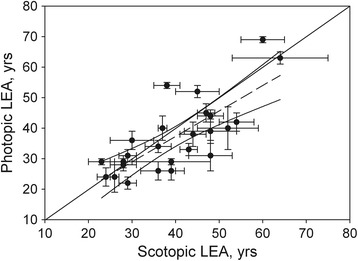
Scatter plot comparing LEA results obtained under photopic and scotopic conditions. The equation of the regression line (dashes) is y = 0.833× + 3.945. The 95 % confidence limits are shown (curved lines). The solid line refers to slope unity

The LEAs obtained from the scotopic and photopic experiments are shown in columns 3 and 4 of Table 1 and in the scatter plot shown in Fig. 6.

**Table 1:** LEAs measured under scotopic (column 3) and photopic (column 4) conditions

Subject #	Age	LEA ± SD (scotopic)	LEA ± SD (photopic)	LEA ± SD (photopic) L:M = 0.47	LEA ± SD (photopic) L:M = 16.5
1	71	60 ± 5	69 ± 1	75 ± 1	66 ± 1
2^a^	34	43 ± 2	33 ± 2	41 ± 2	32 ± 2
3	24	29 ± 2	31 ± 2	39 ± 2	30 ± 2
4	28	37 ± 1	40 ± 4	43 ± 4	34 ± 4
5	28	28 ± 3	28 ± 2	31 ± 1	22 ± 1
6	40	47 ± 2	45 ± 3	49 ± 3	39 ± 3
7	27	52 ± 7	40 ± 7	44 ± 8	34 ± 8
8	40	48 ± 6	39 ± 4	44 ± 5	33 ± 4
9	30	39 ± 9	29 ± 1	37 ± 2	25 ± 2
10^a^	36	48 ± 5	31 ± 5	34 ± 4	25 ± 4
11	60	45 ± 5	52 ± 2	57 ± 2	47 ± 2
12	61	64 ± 11	63 ± 2	68 ± 2	58 ± 2
13	53	48 ± 3	44 ± 2	48 ± 3	40 ± 3
14	28	23 ± 3	29 ± 1	33 ± 1	22 ± 2
15^a^	26	39 ± 3	26 ± 3	29 ± 3	20 ± 3
16	24	36 ± 4	26 ± 3	29 ± 5	20 ± 5
17	36	30 ± 5	36 ± 3	45 ± 2	35 ± 2
18	32	44 ± 3	38 ± 4	42 ± 4	32 ± 4
19	28	26 ± 1	24 ± 5	29 ± 4	18 ± 4
20	29	24 ± 2	24 ± 3	29 ± 4	20 ± 4
21	28	28 ± 3	29 ± 2	33 ± 3	23 ± 2
22^a^	61	38 ± 3	54 ± 1	57 ± 2	47 ± 2
23	46	36 ± 3	34 ± 2	37 ± 2	27 ± 2
24^a^	28	29 ± 2	22 ± 2	28 ± 3	17 ± 3
25^a^	51	54 ± 4	42 ± 3	44 ± 2	33 ± 2

Significant differences are indicated (^a^) in column 1 for 6 subjects. In columns 5 and 6, the LEAs under photopic conditions have been recalculated assuming extreme cone weights of L:M = 0.47 and 16.5, respectively. All ages are in years

**Fig. 7:**
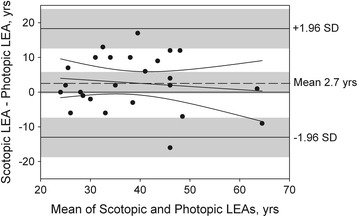
Bland Altman plot for testing the level of agreement between LEAs obtained under photopic and scotopic viewing conditions. The dashed horizontal line represents the mean difference between the scotopic and the photopic LEAs, with the 95 % confidence interval shown in gray. The limits of agreement (mean ± 1.96 SD) are included with their associated 95 % confidence intervals shown in gray. The sloping regression line and its 95 % confidence limits have been added to the plot

The solid line of slope unity represents equality of the two LEA measurements while the dashed line is the regression line of LEA (photopic) on LEA (scotopic). The slope of the regression line was 0.833 ± 0.144 with r^2^ = 0.59, *p* < 0.0001. The intercept was 3.95 ± 5.96 years. A Bland Altman analysis, shown in Fig. 7, was carried out as an additional check for any systematic deviation between the two methods. A Shapiro-Wilk test indicated that the differences met the standard of normality, thereby validating the use of Bland Altman analysis. The mean difference between the scotopic and the photopic LEAs from the analysis was 2.7 years and the limits of agreement (±1.96 SD) were ± 16 years. Also shown in Fig. 7 are the regression line (difference on mean) and the associated 95 % confidence limits.

## Discussion

An overall comparison of the results of the two methods of obtaining the LEA can be found in the slope (0.833 ± 0.144) and intercept (3.95 ± 5.96 years) of the regression line in Fig. 6. The 95 % confidence limits contain the line of equality of LEA by the two methods, indicating no significant difference, on average, between them. Likewise, the Bland-Altman plot of Fig. 7 showed a mean difference of 2.7 years between the LEAs obtained by the two methods, reflecting a small trend of a lower LEA, on average, when measured photopically by Mapcat sf than when it is measured scotopically at absolute threshold. However, the associated 95 % confidence interval (gray area) included the line of equality (0 year) showing the trend was not significant. The regression line added to the Bland-Altman plot (Fig. 7) had a negative slope suggesting that younger subjects had a larger difference (scotopic LEA-photopic LEA) than older subjects. Again the associated confidence interval included the line of equality indicating that the trend was insignificant.

We compared these results with measurements reported by Wooten et al. [12] under photopic and scotopic conditions. The slope of their regression line, lens optical density (photopic) on lens optical density (scotopic), was 0.92, with r^2^ = 0.64, *p* < 0.0002; results which are rather similar to our own LEA results (slope = 0.833 ± 0.144 with r^2^ = 0.59, *p* < 0.0001). They too obtained a positive but insignificant intercept of 0.09 density units that we estimate to be equivalent to about 5 to 6 years in LEA [19]. Again, this is of the same order as our own intercept of ~4 years.

Like those of Wooten et al. [12], our photopic results were slightly lower, on average, than the scotopic ones. However, if we examine our data for individual subjects, significant differences emerge. To determine the significance, we calculated the absolute difference, ∣ scotopic LEA-photopic LEA∣, and the associated standard deviation. We applied a criterion that if the range, difference -2SD to difference +2SD, included zero, the difference was not significant. Accordingly, differences in LEA by the two methods that were not deemed significant were limited to 19 out of the 25 subjects. The remaining 6 subjects are indicated in Table 1 by a superscript a. Further analysis was therefore conducted in an attempt to shed light on the discrepancies.

The standard CIE-approved 10° luminous efficiency function corresponds to an estimate of the average weighting of L and M cones [18]. However, considerable variation in the L:M cone ratio has been reported: 0.6 to 12 by Carroll et al. [34], 1.1 to 16.5 by Hofer et al. [35] and 0.47 to 15.82 by Sharpe et al. [18]. The luminous efficiency function is also affected by L-cone polymorphisms which are common, and M-cone polymorphisms which are rare [18]. However, the effects are small in comparison with variations associated with different L:M ratios. To determine the effect of these variations on the LEAs measured by Mapcat sf, we used the template for the 10° luminous efficiency function proposed by Sharpe et al. [18]. With this template, photopic luminous efficiency functions can be generated for any L:M ratio. Using each subject’s ratio of perifoveal flicker null settings, $\frac{\phi_{BP}}{\phi_G}$ , obtained on Mapcat sf (see Eq. 4), we calculated the LEA as described earlier, but using instead age-modified photopic luminous efficiency functions based on L:M ratios of 0.47, the lower extreme reported by Sharpe et al. [18], and 16.5, the upper extreme reported by Hofer et al. [35]. (Note, however, that the effects on LEA are almost constant for L:M ratios greater than approximately 8.) The results are shown in columns 5 and 6 of Table 1. For the 6 subjects with significant differences between their LEAs by the photopic and scotopic methods, we repeated our analysis of significance but with their photopic LEAs calculated for an extreme cone weight that provided a value closer to the scotopic LEA. With this modification, significant differences were eliminated for 2 of the 6 subjects. The remaining 4 were subjects # 10, 15, 22 and 25. Thus it is conceivable, but unproven, that some, but not all, of the observed differences could be due to variability of cone weights among individuals.

The larger uncertainties in the scotopic measurements, evident from the error bars in Fig. 6, reflect the general impression gained from the subjects that the test was far more difficult than the photopic one. This is one reason for placing greater trust in the photopic measurements. Another reason is to be found in comparisons between the subjects’ biological ages and their LEAs obtained by the two methods. When plotting LEA measured photopically as a function of biological age, the regression line had a slope of 0.79 ± 0.08 and an intercept of 7.3 ± 3.4 years (r^2^ = 0.79, *p* < 0.0001). In the scotopic case, the regression line had a slope of 0.54 ± 0.13 and an intercept of 19.2 ± 5.1 year (r^2^ = 0.44, p = 0.0003). Thus the LEA obtained photopically is generally closer to the subject’s biological age than that obtained scotopically, and is more highly correlated. There are other factors which could potentially influence the scotopic measurements. The apparatus provided a viewing aperture that did not constitute an artificial limiting pupil. Thus, a change in natural pupil size would alter the amount of light entering the eye and this would affect the threshold. Note, however, that this would not be a concern in the photopic method where pupil size changes would affect the two components of the flickering stimulus equally. We have estimated the effect of a potential change in pupil size during the course of scotopic measurements. Pupillometry measurements on dark-adapted adults by Alpern and Campbell [36] and Hansen and Fulton [37] show a short-term variability in pupil diameter. From their data, we have estimated the ratio of maximum to minimum pupil area to be 1.18 and 1.12, respectively, or 1.15 on average. Nevertheless, some of the variability may be due to uncertainty in measurement. Assuming a worst case scenario where, for example, the blue and green stimulus thresholds were determined at maximum and minimum pupil areas, respectively, we have calculated the effect on the LEA to be an overestimate of about 10 years. This becomes an underestimate of about 10 years if the blue and green stimulus thresholds were determined at minimum and maximum pupil areas, respectively. Examination of columns 3 and 4 in Table 1 indicates that the difference between the photopically and scotopically determined LEAs exceeded 10 years in only five subjects. Of these, only subject #10 exhibited a difference that could not be accounted for by a combination of the pupil size variation described here and an extreme L:M cone ratio of 0.47. However, it must be emphasized that pupil size variation, in addition to L:M cone ratio variation, remains speculative, and an unproven reason, as yet, for the differences.

Another factor that we have considered is a potential change in the level of the subject’s dark adaptation during the test. For example, if the subject, in a state of incomplete dark adaptation, began the test with the blue stimulus and concluded, fully dark adapted, with the green stimulus, the LEA would tend to be higher than the true value. However, dark adaptation curves, such as those of Hecht and Mandelbaum [32], indicate that absolute threshold is attained after 30 min, even after initial adaptation to a very bright stimulus. Therefore, we do not believe that incomplete dark adaptation was a significant factor. For the photopic measurements, potential changes in adaptation would also not be a concern owing to the use of a large, uniform visual field surrounding the stimulus and matching its luminance at the flicker null point [1].

## Conclusion

The main goal of this study was to validate LOD measurements made with the Mapcat sf under photopic conditions. The primary purpose of a reliable measurement was to be able to correctly compensate for LOD in the calculation of MPOD that the Mapcat sf performs. A secondary advantage would be to provide optometrists and ophthalmologists with a means of quantifying lens yellowing and monitoring its rate of progression. Since measuring LOD under scotopic conditions, especially at absolute thresholds, requires prolonged periods of dark adaptation, photopic methods are preferable for routine testing. The scotopic measurements revealed no systematic deviations from the photopic measurements on the Mapcat sf, and we suggest that individual differences, when these occur, are more likely to be attributable to the scotopic test.
